# Effect of Same-Temperature GaN Cap Layer on the InGaN/GaN Multiquantum Well of Green Light-Emitting Diode on Silicon Substrate

**DOI:** 10.1155/2013/538297

**Published:** 2013-12-04

**Authors:** Changda Zheng, Li Wang, Chunlan Mo, Wenqing Fang, Fengyi Jiang

**Affiliations:** National Engineering Technology Research Center for LED on Si Substrate, Nanchang University, Nanchang 330047, China

## Abstract

GaN green LED was grown on Si (111) substrate by MOCVD. To enhance the quality of InGaN/GaN MQWs, same-temperature (ST) GaN protection layers with different thickness of 8 Å, 15 Å, and 30 Å were induced after the InGaN quantum wells (QWs) layer. Results show that a relative thicker cap layer is benefit to get InGaN QWs with higher In percent at fixed well temperature and obtain better QW/QB interface. As the cap thickness increases, the indium distribution becomes homogeneous as verified by fluorescence microscope (FLM). The interface of MQWs turns to be abrupt from XRD analysis. The intensity of photoluminescence (PL) spectrum is increased and the FWHM becomes narrow.

## 1. Introduction

Although GaN-based blue/green LEDs have been commercially used in color digital display, liquid crystal display backlighting, traffic lamps, vehicle lamps, and general lighting areas as the significant progress achieved in the material growth and device manufacture, on the way to widely substitute the incandescent and fluorescent lamps as the general lighting source, cost lowing and electricity to light convert efficiency improvement are still required for III-nitride LEDs [1, 2].

Generally estimated, substrates cost 25 to 35 percent of the LED chips. So, how to reduce the cost of substrates is one of the key tasks for researchers. Due to lack of high quality, inexpensive, and large size GaN single-crystal substrate, commercially used GaN LED structures are mostly grown either on sapphire or SiC substrates. But there still exists some deficiency for both substrates. For example; they both are relatively expensive (especially SiC substrates) and hard, which introduces great complexity and trouble to the device fabrication processes and ultimately improves the devices cost. Therefore, silicon is used by some researchers as substrate to grow GaN-based LEDs and reduce device cost for its low cost, availability of large size, high surface quality, high conductivity, and well-established processing techniques [3–5]. Today GaN blue LEDs have already been grown on silicon substrate by several groups, which provide one of the low-cost solutions for solid state lighting [6–8].

Otherwise, the increase in efficiency and output of GaN LEDs is still required. The difficulty to grow high efficient LEDs is mainly due to the fundamental problem associated with the growth of InGaN/GaN MQWs [9, 10]. Especially for green LEDs, high-Indium content InGaN layers with good crystal quality are necessary [11–13]. Hence, on the one hand, InGaN QWs must be gained with homogeneous indium incorporation. On the other hand, InGaN MQWs with abrupt interface are also needed [14–16]. In this study, based on the successful growth of crack free GaN epilayer on silicon substrate [7], we researched the effect of cap layer thickness on the quality of the InGaN QW material and the well/barrier interface.

## 2. Experiment

**Figure 1:**
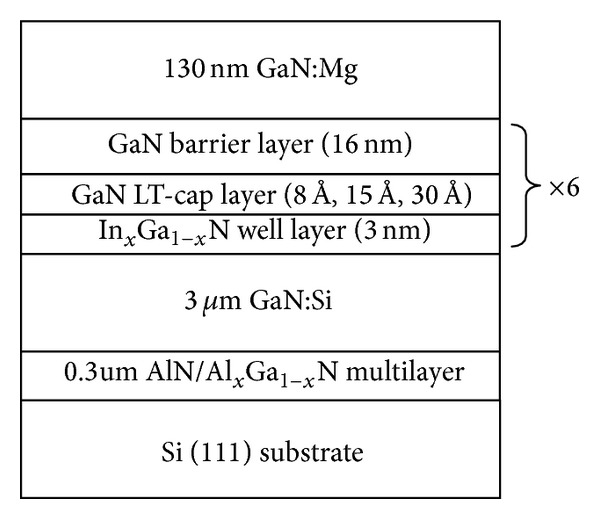
Epilayer structure of green InGaN/GaN light emitting diodes on silicon (111) substrate.

The InGaN/GaN MQWs green LED structure (as shown in Figure 1) was grown on silicon (111) substrate by Thomas Swan 6 × 2′′ MOCVD with close-coupled showerhead reactor. Before being loaded into reactor, silicon substrates were degreased by H_2_SO_4_ : H_2_O_2_ = 3 : 1 mixture solution and etched with diluted HF (5%) solution to remove the surface contamination and oxide layer. After 30 minutes H_2_ in situ heating at 1100°C in reactor, only TMAl carried by H_2_ was firstly injected into reactor for 2 minutes at about 800°C to form a thin Al film on silicon surface and avoid silicon refused by metal gallium and/or nitrificated to SiN by ammonia (NH_3_). A 0.3 *μ*m AlN/Al_*x*_Ga_(1−*x*)_N stress releasing layer and 3 *μ*m GaN:Si layer were then grown. 200 nm InGaN strain prerelief layer and 6 periods InGaN/GaN MQWs were grown on *n*GaN. 130 nm GaN:Mg layer was lastly capped on the MQWs. For MQWs, every period was composed of InGaN well layer, GaN cap layer at well temperature, and GaN barrier layer at temperature 120°C higher than well. The cap layers were designed with three thicknesses of 8 Å, 15 Å, and 30 Å and those samples were marked as sample “A,” sample “B,” and sample “C,” respectively. Double crystal X-ray diffractometry (DCXRD) was used to characterize the structure quality of MQWs. Fluorescence microscope (FLM) was used to get fluorescence image of QWs under the optional excitation wavelength from 380 nm to 420 nm. The selected wavelength of dichroic mirror is 430 nm and barrier filter is 450 nm in this study. Photoluminescence (PL) spectrum was used to characterize the optical performance of InGaN/GaN MQWs excitated with He-Ge 325 nm laser.

## 3. Results and Discussion

**Figure 2:**
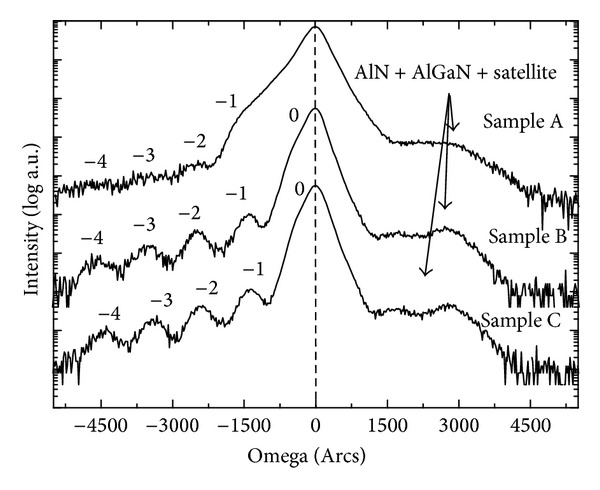
DCXRD Omega-2theta rocking curve on GaN (0002) plan for three samples. The center peak is defined as GaN (002) peak. InGaN diffraction peak is marked as zero order peak.

XRD Omega/2theta rocking scan was induced to check the quality of MQWs. The scan range is from −5000arcsec to +5000arcsec. The diffraction peak of GaN (0002) plan is set on the symmetry scanning center of the rocking curve and is located as 0 position of the *x*-coordinate. As shown in Figure 2, satellite peaks clearly observed for samples B and C on the left side of diffraction curves, indicating that fine QW/QB periodic structure and abrupt QW/QB interface are formed for MQW with 15 Å and 30 Å cap layer. 1st to 4th order satellite peaks are also appeared for these two samples. Still, it is found that 3rd and 4th order peaks widths of sample B are even larger compared with those of sample C, showing that sample B has relative worse QW/QB interface. Furthermore, when the cap thickness is reduced to 8 Å (Sample A), the satellite peaks become not easy to be distinguished from the left side of diffraction curve. This means that clear QW/QB periodic structure was not formed for the well being destroyed when temperature ramping from well to higher barrier temperature with only 8 Å thin cap layer. For samples B and C, an overall peak can be differentiate from the left of GaN (0002) peak, which is assigned to 0 order peak from the well In⁡_*x*_Ga_(1−*x*)_N (0002) diffraction. As the insufficiently resolution of the double crystal X-ray diffractionmeter, this peak is not so clearly and encapsulated in GaN peak with a small signal that appeared on the left shoulder of GaN (0002) peak. For sample A, no 0 order peak can be resolved from the curve. The reason may also be stem from the heat-damage effect and heterogeneous In fraction of QW for sample A. For all three samples, there are no clearly symmetric satellite peaks on the right side of the diffraction curves as which lies on the left side. One reason is that the X-ray intensity decreases as the diffraction angle increases. Another reason is that AlN and AlGaN multilayer was grown to release the stress between Si substrate and GaN layer and the multilayer diffraction peaks are also laid on the right of GaN (002) peak. These peaks superpose with MQWs satellite peak, which brings on an indecipherable overall peak.

**Figure 3:**
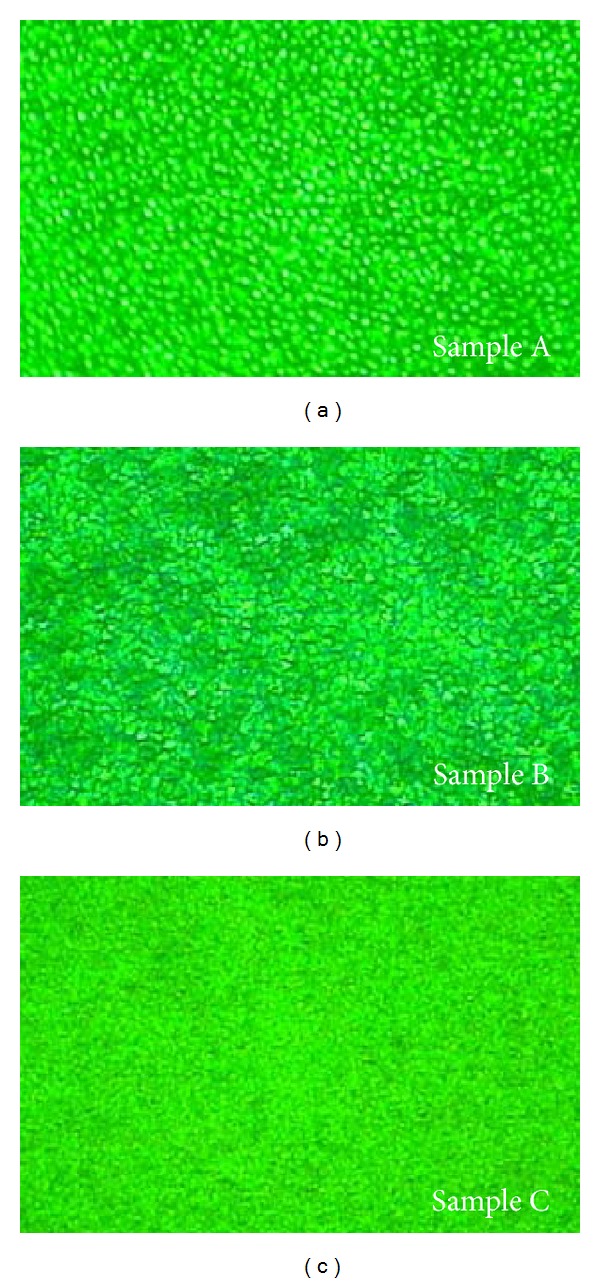
The FLM images for three samples. The selected wavelength of dichroic mirror is 430 nm and barrier filter is 450 nm in the test.

The FLM images of the samples are showed in Figure 3. It is found that sample A has an uneven FL image. Extra-bright points are distributed on the picture. This is caused by the well InGaN decomposed and segregated when ramping to grow barrier and p-GaN at high temperature. For image of sample B, the uneven points become not so serious. No extremely bright points can be found from sample B. But some local uneven areas still exist on the image. When cap thickness increased to 30 Å, the image becomes more uniform and no big bright points appear on it. The FL images are usually used to reflect the Indian distribution in the well. It can be concluded that the MQWs have a much more uniform fluorescence microscope images as increasing the cap layer thickness. Thicker cap would protect the well from being decomposed. The results are also in accordance with the conclusions from XRD test.

**Figure 4:**
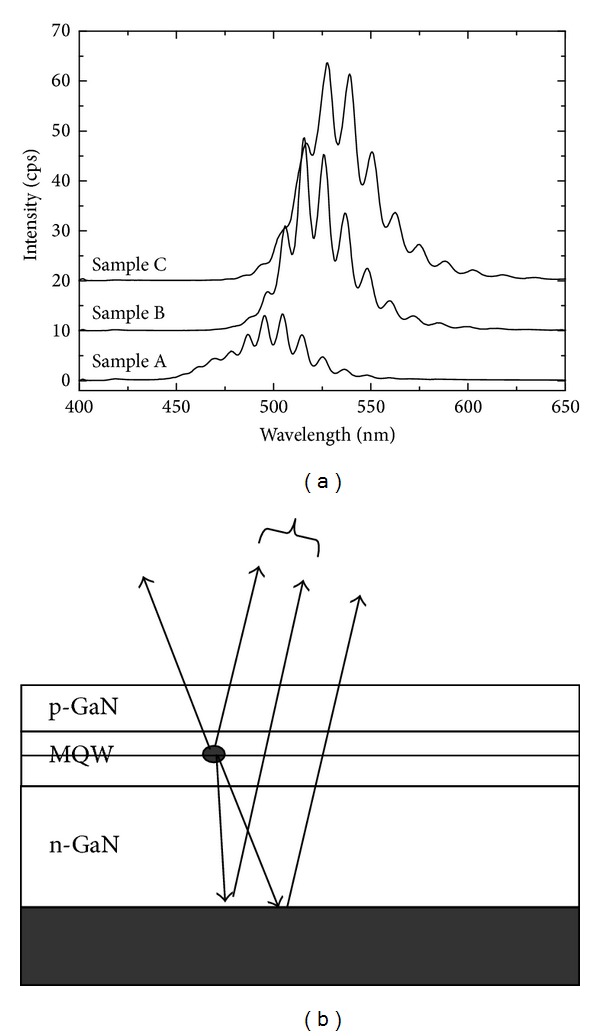
(a) PL spectra of the three samples are excitated by He-Ge 325 nm laser at room temperature. (b) Schematic diagram of optical cavity effects of the MQWs light from the surface and GaN/Si interface reflection.

The lighting characteristic of MQWs was taken by photoluminescence (PL) measurement with He-Ge 325 nm laser as excitated source. The PL spectra of three samples are shown in Figure 4(a). The peak wavelength is all loaded in green wavelength ranged from 500 nm to 535 nm. All the spectra are not smooth curve but have interference candy stripes accompanying with the curve. Those stripes are from optical cavity effects [17]. As shown in Figure 4(b), green light is generated from MQW excitated by laser. Some fraction of light is directly extracted out the wafer surface. Some fraction of light is firstly reflected by GaN/Si interface and then extracted out the wafer surface. The two lights have optical path difference, forming regions of high and low intensities. As the cap increases from 8 Å to 30 Å, the intensity of the PL spectra increases and the peak wavelength is red-shifted from 500 nm to 530 nm. At the same time, the full width of half maximum (FWHM) of the spectra becomes narrow. This can also be explained by the well protection effect leading to increasing the In component and uniformity with the thicker cap layer.

## 4. Conclusions

In conclusion, GaN-based green LED structure with InGaN/GaN MQWs has been grown on Si (111) substrate by MOCVD. InGaN QW was found to decompose at the higher temperature when growing GaN barrier and Mg-doped GaN:Mg layer, which results in heterogeneous indium component in InGaN QW-layer and poor InGaN/GaN interface. Same-temperature GaN cap layer after InGaN QW is effective in preventing the InGaN decomposition. As the cap thickness increases, the indium distributions become homogeneous as verified by FL. The interface of MQWs turns out to be abrupt from XRD analysis. The intensity of PL spectrum is increased and FWHM becomes narrow. Thus, the cap layer is one of the key optional tuning parameters to improve GaN-based green MQWs quality and further to obtain high-efficiency LEDs.
